# Shear Bond Strengths of Methacrylate- and Silorane-based Composite Resins to Feldspathic Porcelain using Different Adhesive Systems

**DOI:** 10.15171/joddd.2015.033

**Published:** 2015-09-16

**Authors:** Narmin Mohammadi, Maryam Shakur Shahabi, Soodabeh Kimyai, Fatemeh Pournagi Azar, Mohammad Esmaeel Ebrahimi Chaharom

**Affiliations:** ^1^Associate Professor, Department of Restorative Dentistry, Faculty of Dentistry, Tabriz University of Medical Sciences, Tabriz, Iran; ^2^Postgraduate Student, Department of Restorative Dentistry, Faculty of Dentistry, Tabriz University of Medical Sciences, Tabriz, Iran; ^3^Professor, Department of Restorative Dentistry, Faculty of Dentistry, Tabriz University of Medical Sciences, Tabriz, Iran; ^4^Associate Professor, Department of Restorative Dentistry, Faculty of Dentistry, Tabriz University of Medical Sciences, Tabriz, Iran

**Keywords:** Adhesive, bond strength, porcelain, silorane

## Abstract

***Background and aims.*** Use of porcelain as inlays, laminates and metal-ceramic and all-ceramic crowns is common in modern dentistry. The high cost of ceramic restorations, time limitations and difficulty of removing these restorations result in delays in replacing fractured restorations; therefore, their repair is indicated. The aim of the present study was to compare the shear bond strengths of two types of composite resins (methacrylate-based and silorane-based) to porcelain, using three adhesive types.

***Materials and methods.*** A total of 156 samples of feldspathic porcelain surfaces were prepared with air-abrasion and randomly divided into 6 groups (n=26). In groups 1-3, Z250 composite resin was used to repair porcelain samples with Ad-per Single Bond 2 (ASB), Clearfil SE Bond (CSB) and Silorane Adhesive (SA) as the bonding systems, afterapplication of silane, respectively. In groups 4-6, the same adhesives were used in the same manner with Filtek Silorane composite resin. Finally, the shear bond strengths of the samples were measured. Two-way ANOVA and post hoc Tukey tests were used to compare bond strengths between the groups with different adhesives at P<0.05.

***Results.*** There were significant differences in the mean bond strength values in terms of the adhesive type (P<0.001). In addition, the interactive effect of the adhesive type and composite resin type had no significant effect on bond strength (P=0.602).

***Conclusion. ***The results of the present study showed the highest repair bond strength values to porcelain with both composite resin types with the application of SA and ASB.

## Introduction

Use of porcelain as inlays, onlays, laminates and metal-ceramic and all-ceramic crowns is common in modern dentistry. Feldspathic porcelains or leucite-based glass-matrix dental ceramics are commonly used due to their high esthetic appearance.^1^

Factors such as fatigue, occlusal forces, inappropriate design, poor preparation of the abutment and technical errors during laboratory procedures and physical traumas can result in the failure of cohesive bonds in the porcelain.^2,3^ The high cost of ceramic restorations, time limitations and difficulty of removing these restorations result in delays in replacing fractured restorations; therefore, their repair is indicated.^4^ The surface of ceramics should be prepared by roughening with the use of rotary diamond burs, by abrasion using air-borne particles, and by surface etching by hydrofluoric acid (HF) or acidulated phosphate fluoride (APF) gel. When etching by HF is supplemented with the use of air-borne particles and silane, the bond strength of composite resin to different types of ceramic increases significantly.^5^

An important factor to increase adaptation of composite resin to the surface of ceramic is the use of an interfacial bonding agent.^6^ At present, various adhesive systems, including self-etch and etch-and-rinse systems, are available. These adhesives might be methacrylate-based or of the newer silorane-based type.^7^ Silorane Adhesive Systems, marketed as a self-etch primer and bonding agent, are new members of self-etch adhesives.^8^ The conventional chemical basis for all the restorative composite resins is the polymerization of methacrylate or acrylate radicals; however, in restorative silorane systems, a combination of two chemical structures of siloxane and oxirane provides a hydrophobic base with low shrinkage for silorane-based restorative materials. This new resin matrix is the main difference between silorane-based restorative materials and conventional methacrylate-based ones. In general, the ring-opening polymerization system results in a decrease in polymerization shrinkage, leading to lower shrinkage forces at margins.^8^

Different studies have evaluated the bond strength of composite resin to porcelain, using different preparation techniques for porcelain surfaces. In a recent study, Ozcan evaluated the bond strength of composite resin to alumina-reinforced ceramic with the use of different repair systems, followed by thermocycling and storage in water and reported that Cojet system exhibited higher bond strength under such conditions.^1^ In a study different porcelain repair systems were compared. The results showed higher bond strength using surface roughening with Cojet system (sandblasting with SiO_2_) compared to Ceramic Repair, Cimara and Clearfil Repair Systems.^9^ El Zohairy et al evaluated the effect of adhesive systems on the durability of the bond between the ceramic and resin and showed higher durability of the bond achieved with the use of hydrophobic bonding agents after storage in water.^6^

Since no studies to date have evaluated the efficacy of silorane-based composite resins and adhesives on the repair of porcelain, the aim of the present study was to compare the shear bond strength of different adhesive systems used for the repair of porcelain with the use of relevant composite resins. The null hypothesis was that there are no significant differences in shear repair bond strengths of various adhesives to feldspathic porcelain with two types of composite resins.

## Materials and Methods

A total of 156 feldspathic porcelain samples (Ceramco, Dentsply, USA), measuring 5 mm in height and diameter, were prepared in a porcelain oven (VITA VACUMAT 40 T, VITA Zahnfabrik, Germany) based on manufacturer’s instructions. The surface of each porcelain sample was polished under water cooling, using 600-grit silicon carbide paper (3M ESPE, St. Paul, MN, USA).

An intraoral air abrasion device (Microblast, Dental, Microblaster, Denmark) was used to create surface roughness in all the samples, using 50-µ aluminum oxide particles at a pressure of 50 bar, with the device tip placed at a distance of 10 mm from the sample surface for 10 seconds.^10^ Then the roughened samples were mounted in acrylic blocks with 2 mm of the porcelain out of the acrylic resin in order to facilitate application of the shearing force in the relevant machine. At this stage, the samples were randomly divided into 6 groups (n=26).

In group 1, the porcelain surfaces were exposed to 37% phosphoric acid for 2 minutes^6^ and then dried. At this stage, the interfacial silane material (Pulpdent, Watertown, USA) was applied on the dried surface for 60 seconds and air-dried.^6^ Then a layer of the methacrylate-based Adper Single Bond 2 (3M, ESPE, Dental Product, St. Paul, MN, USA) etch-and-rinse adhesive was applied. After application of a mild jet of air with an air-and-water syringe, light-curing was carried out with Astralis 7 (Ivocular Vivadent, AG, FL-9494, Schaann, Liechtenstein) QTH light-curing unit (at a light intensity higher than 400 mW/cm^2^) for 20 seconds. Then Z250 methacrylate-based composite resin (3M, ESPE, Dental Products, St. Paul, MN, USA) was placed in bulk on the porcelain surface using plastic molds measuring 2 mm in height and diameter and light-cured for 40 seconds.

In group 2, the roughed porcelain surfaces were silanized in a manner similar to that in group 1. Then the primer and bonding agent of Clearfil SE Bond (Kuraray Medical INC, Osaka, Japan) methacrylate-based self-etch system were applied to porcelain surfaces according to manufacturer’s instructions and light-cured for 20 seconds. Z250 composite resin was placed on porcelain surfaces in a manner similar to that in group 1.

In group 3, the samples were silanized similar to that in group 2 and the primer and bonding of Silorane Adhesive System (3M, ESPE, Dental Products, St. Paul, MN, USA) were applied according to manufacturer’s instructions and Z250 composite resin was placed on porcelain surfaces similar to that in group 1.

The samples in groups 4-6 were repaired similar to the procedures used in groups 1-3 with the use of Filtek Silorane silorane-based composite resin, respectively. All the samples were immersed in distilled water at 37°C for 7 days and underwent a 500-cycle thermocycling procedure at 5±2/55±2°C with a dwell time of 30 seconds and a transfer time of 10 seconds.^11^ A detailed description of the materials used in study is provided in Table 1.

**Table 1:** Materials used

Material	Composition	Manufacturer
Ceramco Feldspathic porcelain	SiO_2_ 60%, Al_2_O_3_ 20% Na_2_O ,K_2_O, B_2_O_3,_ ZnO	Dentsply, USA
Adper Single Bond 2	HEMA, water, ethanol, amines, Bis-GMA, methacrylate-functional, copolymer of polyacrylic and polyitaconic acids, dimethacrylates.	3M ,ESPE, Dental Products St Paul, MN, USA
Clearfil SE Bond	*Primer:* MDP, HEMA, hydrophilic aliphatic dimethacrylate, dicamphoroquinone, N-diethyl-p-toluidine, and water *Bond:* MDP, bis-GMA, HEMA, hydrophobic aliphatic dimethacrylate, dicamphoroquinone, N-diethyl-p-toluidine, and colloidal silica	Kuraray Medical INC, Osaka, Japan
Silorane Adhesive System	*Primer:* 15–25% HEMA; 15–25% Bis-GMA); 10–15% water; 10–15% ethanol; 5–15% phosphoric acid–methacryloxy– hexylesters; 8–12% silane treated silica; 5–10% 1,6-hexanediol dimethacrylate; <5% copolymer of acrylic and itaconic acid; <5% (dimethylamino) ethyl methacrylate; <3% dl-camphorquinone; <3% phosphine oxide *Bond:* 70–80% substituted dimethacrylate; 5–10% silane treated silica; 5–10% TEGDMA; <5% phosphoric acid–methacryloxy–hexylesters; <3% dl-camphorquinone; <3% 1,6-hexanediol dimethacrylate	3M ,ESPE, Dental Products St Paul, MN, USA
Silane Bond Enhancer	Silane, 1–3% , Ethanol, 92.6%; acetone, 7.4%	Pulpdent, Watertown, USA
Filtek Silorane	5–15% 3,4-epoxycyclohexylethylcyclopolymethylsiloxane; 5–15% bis-3,4-poxycyclohexylethylphenylmethylsilane; 50–70% silanized quartz;10–20% yttriumfluoride; camphorquinone	3M,ESPE, Dental Products St Paul, MN, USA
Filtek Z250	Bis-GMA, UDMA, Bis-EMA, camphorquinone, 60% filler	3M,ESPE, Dental Products St Paul, MN, USA
Abbreviations:		
Bis-GMA: bisphenol a diglycidyl ether dimethacrylate		
MDP: 10-Methacryloyloxydecyl dihydrogen phosphate		
HEMA: 2-hydroxyethyl methacrylate		
TEGDMA: triethylene glycol dimethacrylate		
UDMA: Urethane dimethacrylate		

All the samples in acrylic molds underwent a shearing force at a crosshead speed of 1 mm/min in a universal testing machine (Hounsfield Test Equipment, Model H5K-S, England) in order to measure the shear bond strength of composite resin to porcelain by placing the chisel-shaped blade of the machine at the composite resin-porcelain interface. The bond strength values were calculated in MPa by dividing the bond strength values measured in Newton by the bonded surface areas of samples in mm^2^.

After the fracture of the samples, failure modes were determined under a stereomicroscope (SMZ 1500, Nikon, Japan) based on the following classification:^1^

Adhesive failure: fracture at composite resin‒porcelain interface

Cohesive failure: fracture within the composite resin or porcelain

Mixed failure: a combination of the two above

Data were analyzed with descriptive statistical methods using SPSS 15/Win. Two-way ANOVA and post hoc Tukey tests were used to compare bond strengths between the groups with different adhesives at P<0.05.

## Results

Table 2 presents descriptive data of porcelain repair bond strength values and the results of statistical comparisons between the study groups.

**Table 2:** Means and standard deviations (SD) of the repair bond strength values (in MPa) in the study groups

Composite resin	Adhesive system	mean	SD	N
Methacrylated-based composite resin (Z250)	Adper Single Bond 2	19.18^a^	9.11	26
	Clearfil SE Bond	10.48^b^	8.95	26
	Silorane Adhesive	21.33^a^	6.66	26
Silorane-based composite resin (Filtek ‏ Silorane)	Adper Single Bond 2	17.64^a^	7.33	26
	Clearfil SE Bond	11.63^b^	5.98	26
	Silorane Adhesive	19.78^a^	8.57	26
a,b - There is no significant statistic differences between similar characters.		

**Figure 1.**
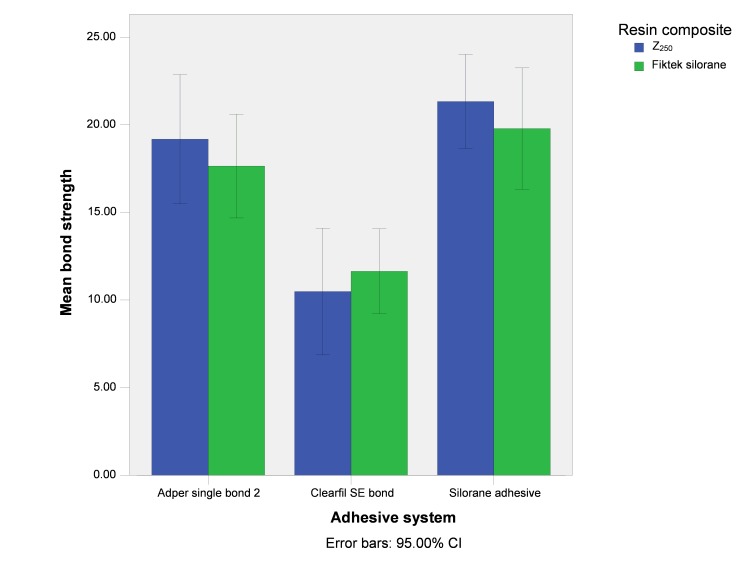
1. Error bar graph related to mean bond strength of composite to porcelain, according to the type of adhe-sive system./p>

The graph in Figure 1 presents the error bars of mean repair bond strength values in the study groups in terms of the adhesive system type.

The results of two-way ANOVA showed significant differences in the mean bond strength values between the different adhesive systems (F_2,150_=20.866; P<0.001).

Based on the results of post hoc Tukey tests there were significant differences in bond strengths between the groups bonded with Adper Single Bond 2 and Clearfil SE Bond adhesive systems (P<0.001), with higher repair bond strength values with the use of Adper Single Bond 2 adhesive system. In addition, there were significant differences in bond strengths between the groups bonded with Clearfil SE Bond and Silorane Adhesive Systems (P<0.001), with higher repair bond strength values with the use of Silorane Adhesive System. No significant differences were observed between the groups bonded with Adper Single Bond 2 System and those bonded with Silorane Adhesive (P=0.348). The mean repair bond strength values were not significantly different in terms of the type of the composite resin (F_1,150_=0.262; P=0.61). In addition, the cumulative effect of the adhesive type and composite resin type had no significant effect on bond strength (F_1,150_=0.509; P=0.602).

Table 3 presents the frequencies of different failure modes. The most frequent fracture mode with the use of Clearfil SE Bond was adhesive and the most frequent fracture mode in Silorane Adhesive and Adper Single Bond 2 Adhesive Systems was mixed. Cohesive failure mode was observed with the use of all the three adhesive systems.

**Table 3:** Frequency of fracture patterns in study groups

**Resin composite**	**Adhesive system**	**Adhesive fracture**	**Mixed fracture**	**Cohesive fracture**	**N**
**Methacrylated based resin composite (Z250)**	Adper Single bond 2	‏8	‏15	3	26
	Clearfil SE bond	21	‏3	‏2	26
	Silorane adhesive	‏7	14	5	26
**Silorane based composite (Filtek Silorane)**	Adper Single bond 2	‏9	‏11	‏6	26
	Clearfil SE bond	‏21	‏5	0	26
	Silorane adhesive	‏12	13	1	26

## Discussion

The present study was undertaken to evaluate the effect of three adhesive types on the repair bond strength of feldspathic porcelain with composite resin; one of these adhesives has recently been introduced for application with the silorane-based low-shrinkage Filtek Silorane composite resin and no studies to date have evaluated the bond strength of this adhesive in the repair of porcelain.

In the present study, air abrasion with 50-µ aluminum oxide particles was used to create surface roughness in porcelain; some previous studies have shown the efficacy of this technique in roughening the surface of porcelain compared to other techniques.^5,12,13^ This surface roughening technique can be used with different kinds of ceramics, contrary to etching with HF in which the presence of a glass phase in the porcelain is necessary to dissolve it and create micromechanical retention.^12^

In all the study groups, silane was used as an interfacial material, which is bonded through a chemical reaction to hydrolyzed silicon carbide groups on the ceramic surface on one side and the methacrylate groups of the resin adhesive on the other.^2,14^ Silane contributes to the bond between the composite resin and the surface of ceramic, which is mediated by siloxane bonds.^15^ On the other hand, use of air abrasion as a technique for roughening the surface increases the surface energy of the porcelain substrate, providing better acceptance for the hydrophilic groups of silane, resulting in better wetting of the surface with silane and increased absorption of hydrophobic adhesives to the hydrophobic groups of silane.^6,15^

In the present study, the highest repair bond strength value was recorded in the samples repaired with Silorane Adhesive, with no significant differences from the samples repaired with Adper Single Bond 2. However, the bond strength in samples repaired with Silorane Adhesive was significantly higher than that in samples repaired with Clearfil SE Bond. In a study by El Zohairy et al^6^ the effects of different bonding agents with different hydrophilicity on the durability of the bond between composite resin and ceramic were evaluated. The results showed that Visio Bond adhesive exhibited a higher durability after predetermined intervals of water storage due to its high hydrophobicity. We used silorane adhesive in the present study by some similarities in hydrophobic properties with Visio Bond adhesive used in El Zohairy et al study. Silorane Adhesive is composed of a self-etching hydrophilic acidic primer (pH=2.7); it is a hydrophobic viscous adhesive. Its adhesive resin content consists of substituted dimethacrylate, triethyleneglycol-dimehacrylate (TEGDMA), silica, camphorquinone and a low concentration of functional monomers, the high hydrophobicity of which is attributed to dimethacrylate.^16^ It has been shown that the hydrophobicity of the adhesive layer is related to the durability of the bond of this adhesive and the hydrophobic covering of Silorane Adhesive provides greater stability for the adhesive layer in the face of water absorption.^17^ In a study by Weinmann et al,^18^ too, Silorane Adhesive exhibited a higher hydrolytic stability compared to methacrylate-based adhesives. Therefore, the high bond strength of Silorane Adhesive might be attributed to its hydrophobic property and viscosity. In the present study, water storage at 37°C was carried out and all the samples underwent thermocycling before the bond strength test in all the groups, similar to a study by El Zohairy et al.^6^

In our study, the samples bonded with Adper Single Bond 2 adhesive exhibited a high bond strength similar to those bonded with Silorane Adhesive, which might be attributed to the hydrolytic stability of this adhesive.^19^ This adhesive consists of Vitre Bond copolymer in a water/ethanol solvent and is resistant to moisture; in fact it is a functional methacrylate copolymer of polyacrylic and polyitaconic acid. It has been shown that incorporation of polyalkenoic acid into the structure of this adhesive contributes to its resistance against destructive effects of moisture.^20^ In a study by Dantas et al,^19^ the effect of water storage on the bond strength of various adhesives was evaluated. The results showed favorable behavior of Adper Single Bond 2 adhesive in preserving its bond stability in a destructive environment. In a study the disintegration of resin‒dentin bond was evaluated in vitro. The disintegration of Adper Single Bond 2 bond to dentin was attributed to the role of the reaction of hydroxyethylmethacrylate (HEMA) with residual water molecules in dentin and formation of poly-HEMA hydrogel which decreases the hydrolytic stability of the bond.^21^ Given the absence of a dentin substrate in the present study and the absence of any moisture in the ceramic substrate, higher stability and bond strength can be expected with the use of Adper Single Bond 2 for the repair of porcelain.

The lowest shear bond strength in the present study was recorded in porcelain samples bonded with Clearfil SE Bond, consistent with the results of studies by dos Santos,^4^ Ozcan^1^ and Blum.^9^ It appears the low bond strength in this group is due to the hydrophilic nature of Clearfil SE Bond adhesive. Clearfil SE Bond contains polymers consisting of hydrophilic monomers such as HEMA which allow absorption of water and failure of the bond.^6^

In the present study, the mean bond strengths in terms of the composite type were not significantly different. Both Filtek Silorane and Z250 composite resins are microhybrid. It has been demonstrated that the type of composite resin can influence the repair bond strength of porcelain and composite resins with bigger particle sizes or hybrid resins exhibit higher bond strengths compared to composite resins with smaller filler size at composite resin‒porcelain interface.^22^

Several studies^9,23-27^, have determined the bond strength of composite resin to feldspathic porcelain and bond strength values different from those of the present study have been reported, which can be attributed to differences in methodologies, including use of different surface preparation techniques, use of different adhesive systems and presence or absence of laboratory simulations of the oral cavity conditions. On the other hand, use of different bond strength evaluation techniques (shear, microshear and microtensile) in different studies^25,28,29^ makes it impossible to directly compare those studies with the present study.

In the present study, the highest bond strength values were recorded with Silorane Adhesives and Adper Single Bond 2. It is suggested that in studies in future the bond strength of new Silorane Adhesive be evaluated in the repair of other ceramics with composite resin. In addition, it is suggested that artificial saliva be used to simulate the oral cavity conditions and the samples be subjected to longer thermocycling procedures and load cycling.

## Conclusion

The efficacy of Silorane Adhesive was comparable to that of total-etch Adper Single Bond 2 adhesive in the repair of feldspathic porcelain with composite resin and higher than that of self-etch Clearfil SE Bond adhesive. Composite resin type had no effect on the repair bond strength of porcelain.
